# PB.16. Breast screening mammograms: recall or not to recall. What is the golden ratio?

**DOI:** 10.1186/bcr3738

**Published:** 2014-11-03

**Authors:** AR Sever, R Pietrosanu, J Weeks, P Mills

**Affiliations:** 1 University of Hacettepe, Ankara, Turkey; 2 Maidstone Hospital, Maidstone, UK

## Introduction

The accuracy of prospectively categorised screening mammograms prior to assessment was evaluated and the individual recall and cancer detection rates for each category were studied.

## Methods

The screening mammograms of women who attended the National Health Service Breast Screening Program over a period of 5 years (April 2008-March 2013) were included in the study. The recalled patients' films were prospectively categorised into one of six groups: patients with normal mammograms requiring clinical recall; probably benign; probably benign/suspicious; suspicious; suspicious/malignant; and malignant. Recalled patients were subsequently assessed with additional mammographic views, ± ultrasound and/or needle biopsy. The recall and cancer detection rates of each category were individually calculated.

## Results

A total of 86,405 mammograms (17,881 prevalent and 68,524 incident rounds) were read and 4,125 patients were recalled for assessment (overall recall rate = 4.8%). A total of 721 patients were diagnosed with cancer (0.8%). The prevalent recall rate was 9.1% (recommended programme target <7%) and incident recall rate was 3.6% (target <5%).

## Conclusion

Mammograms showing minimal signs of cancer that were categorised as 'probably benign' comprised the largest recall group with a relatively low detection rate (7%). If patient recall was limited to only more suspicious groups (that is, PB/suspicious and above), the overall recall rate would have dropped to 1% from 4.8%; however, 28% of the cancers would have missed. Patient anxiety generated due to false positive recalls and the cost of assessment clinics needs to be carefully balanced against a higher cancer detection rate.

